# T-5 Standardizing ROM Assessment in Burn Rehabilitation: Establishing Benchmarks and Red Flag Criteria for Contracture Prevention

**DOI:** 10.1093/jbcr/irag033.005

**Published:** 2026-04-06

**Authors:** Audrey M O'Neil, Renée Warthman, Bethany Murrell, Laura Griffard, Mackenzie Herrmann, Cassandra Rush, Kevin N Foster, Leigh J Spera

**Affiliations:** Richard M Fairbanks Burn Center, Indianapolis, IN; Diane & Bruce Halle Arizona Burn Center - Valleywise Health, Phoenix, AZ; Eskenazi Health, Indianapolis, IN; Eskenazi Health, Indianapolis, IN; Richard M Fairbanks Burn Center, Indianapolis, IN; Eskenazi Health, Indianapolis, IN; Diane & Bruce Halle Arizona Burn Center - Valleywise Health, Phoenix, AZ; Indiana University/Eskenazi Health, Indianapolis, IN

## Abstract

**Introduction:**

Range of motion (ROM) is the primary objective measure in burn care for evaluating and preventing contractures. While standards exist for scar cutaneokinematics, guidelines for contracture assessment remain inconsistent. Severity classifications often divide total ROM into thirds without considering function, and “functional ROM” definitions rarely translate to practice. This project aimed to establish a ROM guidance protocol with performance markers to help clinicians assess ROM, prevent contractures, and manage limitations within functional parameters.

**Methods:**

A literature review was conducted on functional ROM, burn scar contracture severity, and gait mechanics. Norms were identified for the neck, shoulder, forearm, wrist, metacarpophalangeal joints, thumb, hip, knee, and ankle, then reviewed by experienced burn therapists (8–20 years of practice), including certified Burn and Hand Therapy specialists. “Red flag” ranges were defined for each joint with treatment recommendations to address limitations and prevent progression.

**Results:**

The project produced a standardized ROM protocol with benchmarks for prevention and management of burn-related contractures. Guidelines established consistent measurement practices using scar goniometry where applicable and cutaneokinematics, with recommended timing at evaluation, weekly reassessment, and discharge. Yellow flag criteria were identified as early indicators of risk, including ≥10° ROM loss, inability to achieve full ROM for three days, or noncompliance with orthotics or positioning. Each was paired with interventions such as stretching, increased therapy frequency, or orthotics. Red flag thresholds (Table 1) were defined for major joints (e.g., shoulder ≤135°, elbow ≥–20°, knee ≤85°, ankle ≤0°) and linked to targeted actions including orthoses, positioning, BID therapy, strengthening, and functional activities. The protocol incorporated revised goniometric positioning tailored to burn scar patterns, improving measurement reliability. By emphasizing functional relevance, it supported orthotic use and home exercise programs while aligning ROM goals with daily activities. Collectively, the system reduces variability across clinicians, supporting consistent monitoring and reporting.

**Conclusions:**

This protocol provides clear, reproducible benchmarks for contracture prevention and management. Integrating evidence-based norms with expert consensus addresses inconsistencies while supporting earlier detection and intervention. Broader adoption may enhance standardization, improve outcomes, and strengthen comparability across burn rehabilitation programs.

**Applicability of Research to Practice:**

This protocol offers benchmarks and red flags to help clinicians detect contracture risk early and intervene effectively.

**Funding for the Study:**

N/A.

